# Biocontrol of Wheat Crown Rot Using *Bacillus halotolerans* QTH8

**DOI:** 10.3390/pathogens11050595

**Published:** 2022-05-18

**Authors:** Shen Li, Jianqiang Xu, Liming Fu, Guohui Xu, Xiaomin Lin, Junqing Qiao, Yanfei Xia

**Affiliations:** 1 Department of Forensic Medicine, Affiliated Hospital of Guilin Medical University, 15 Lequn Road, Guilin 541001, China; lishen04@stu.haust.edu.cn (S.L.); ghxu2202@glmc.edu.cn (G.X.); 2 Department of Plant Protection, College of Horticulture and Plant Protection, Henan University of Science and Technology, 263 Kaiyuan Avenue, Luoyang 471023, China; xujq@haust.edu.cn (J.X.); 151419030206@stu.haust.edu.cn (L.F.); hlsyyz@haust.edu.cn (X.L.); 3 Institute of Plant Protection, Jiangsu Academy of Agricultural Sciences, 50 Zhongling Street, Nanjing 210014, China

**Keywords:** biocontrol, *Fusarium pseudograminearum*, *Bacillus halotolerans* QTH8, culture filtrate

## Abstract

*Fusarium pseudograminearum* causes crown rot in wheat. This study aimed to assess the effects of the bacterial strain QTH8 isolated from *Cotinus coggygria* rhizosphere soil against *F. pseudograminearum*. Bacterial strain QTH8 was identified as *Bacillus halotolerans* in accordance with the phenotypic traits and the phylogenetic analysis of 16S rDNA and *gyrB* gene sequence. Culture filtrates of bacterial strain QTH8 inhibited the mycelial growth of *F. pseudograminearum* and resulted in mycelial malformation such as tumor formation, protoplast condensation, and mycelial fracture. In addition, bacterial strain QTH8 also inhibited the mycelial growth of *Hainesia lythri*, *Pestalotiopsis* sp., *Botrytis cinerea*, *Curvularia lunata*, *Phyllosticta theaefolia*, *Fusarium graminearum*, *Phytophthora nicotianae*, and *Sclerotinia sclerotiorum*. The active compounds produced by bacterial strain QTH8 were resistant to pH, ultraviolet irradiation, and low temperature, and were relatively sensitive to high temperature. After 4 h exposure, culture filtrates of bacterial strain QTH8—when applied at 5%, 10%, 15%, 20%, 25%, and 30%—significantly reduced conidial germination of *F. pseudograminearum*. The coleoptile infection assay proved that bacterial strain QTH8 reduced the disease index of wheat crown rot. In vivo application of QTH8 to wheat seedlings decreased the disease index of wheat crown rot and increased root length, plant height, and fresh weight. Iturin, surfactin, and fengycin were detected in the culture extract of bacterial strain QTH8 by matrix-assisted laser desorption ionization time-of-flight mass spectrometry (MALDI-TOF-MS). Bacterial strain QTH8 was identified for the presence of the *ituC*, *bacA*, *bmyB*, *spaS*, *srfAB*, *fend*, and *srfAA* genes using the specific polymerase chain reaction primers. *B. halotolerans* QTH8 has a vital potential for the sustainable biocontrol of wheat crown rot.

## 1. Introduction

Wheat crown rot, a worldwide soil-borne disease, is caused by three main pathogens such as *Fusarium pseudograminearum*, *F. graminearum*, and *F. culmorum* [1,2]. Because of its strong pathogenicity and rapid transmission, *F. pseudograminearum* has gradually become the key pathogen [3,4]. Although chemical fungicides constitute a rapid and effective management method, the use of some chemical agents has resulted in problems, such as environmental pollution, human health hazards, and pesticide residues [5]. Therefore, it is urgent to explore novel biological fungicides for managing wheat crown rot.

Biocontrol possesses the advantages of being environmentally friendly, harmless to humans and animals, extensive sources, and has become a new hotspot in plant disease control. The microbial populations currently applied for biocontrol are fungi, bacteria, actinomycetes, etc. Among bacteria, several *Bacillus* spp. have shown antagonistic potential against plant pathogens [6]. For example, *B. subtilis* has been documented to control several plant diseases, such as muskmelon wilt, rice sheath blight disease, crown and root rot of tomato, corn head smut, and verticillium wilt of cotton [7,8,9,10,11]. *B. pumilus* LX11 isolated from peanut rhizosphere showed strong antimicrobial activity against peanut southern blight caused by *Sclerotium rolfsii* [12]. Kong et al. (2010) [13] reported that *B. megaterium* from the Yellow Sea of eastern China significantly controlled a disease caused by *Aspergillus flavus* in peanut kernels. *B. halotolerans* controlled the root rot disease of common bean and pea, verticillium wilt of cotton, grey mold disease of strawberry, and plant-parasitic nematodes of tomato [14,15,16,17,18,19]. These *Bacillus* species produce various antimicrobial compounds, including lipopeptides (LPs), bacteriocins, polyketides, and volatile substances [20]. Of these, lipopeptide antibiotics play a crucial role in disease suppression due to their structural diversity, stable physicochemical properties, a broad spectrum of inhibition, induced systemic resistance, good antimicrobial activity against diseases caused by phytopathogenic fungi, bacteria, etc. [21,22,23]. According to their structures, LPs are generally classified into surfactins, iturins, and fengycins [24,25]. At present, the antimicrobial peptide gene markers of *Bacillus* species are *bacA*, *bmyB*, *bmyC*, *fenA*, *fenD*, *ituA*, *ituC*, *ituD*, *spaS*, *srfAA*, *srfAB*, and so on. Isabel et al. (2011) reported that most *Bacillus* strains have between two and four antimicrobial peptide biosynthesis genes, strains with five of these genes are seldom found, and none of the strains has six or more of these genes [26].

Thus, the present study aimed to: (I) identify the species of bacterial strains isolated from *Cotinus coggygria* rhizosphere soil; (II) assess the potential effects of bacterial strain QTH8 against *F. pseudograminearum*; (III) detect the lipopeptide antibiotics produced by bacterial strain QTH8 and the antimicrobial peptide biosynthetic genes present.

## 2. Results

### 2.1. Antagonistic Effect of Bacterial Strains on Wheat Crown Rot

**Figure 1:**
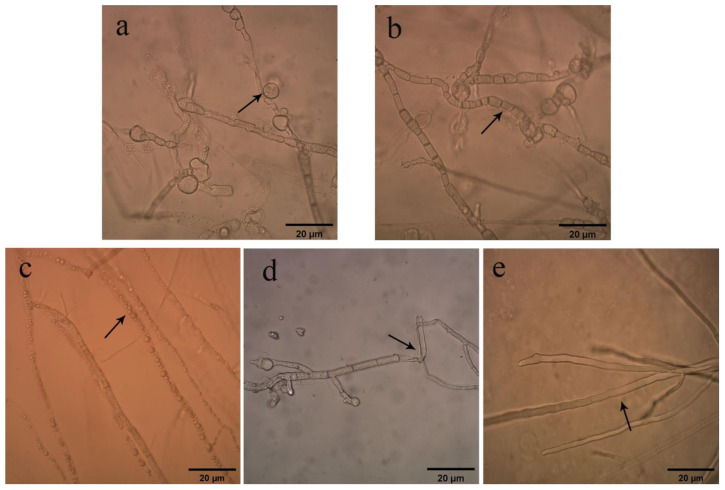
Effects of culture filtrate of bacterial strain QTH8 on mycelia of *Fusarium pseudograminearum*. (**a**) Tumor formation; (**b**) shortening of mycelial septum intervals; (**c**) protoplast condensation; (**d**) crumpled and broken mycelium; (**e**) normal mycelium.

Ten bacterial isolates were recovered from *C. coggygria* rhizosphere soil according to colony morphology. Of these, five strains—which were named QTH1, QTH2, QTH5, QTH7, and QTH8, respectively—showed antagonistic activity against *F. pseudograminearum*. Culture filtrates of bacterial strain QTH8 presented greater antimicrobial activity than the other strains (Table 1). Under the light microscope, mycelia treated with QTH8 showed tumor formation (Figure 1a), shortening of mycelial septum intervals (Figure 1b), protoplast condensation (Figure 1c), and crumpled and broken mycelia (Figure 1d). However, the control mycelia were smooth and uniform (Figure 1e).

### 2.2. Determination of Antimicrobial Spectrum

Next, the effect of bacterial strain QTH8 against eight different phytopathogenic fungal pathogens was assessed using a dual-culture method. QTH8 significantly inhibited *H. lythri* compared with the other seven fungal isolates tested, and its inhibition diameter was 34.09 mm (Table 2). The inhibition diameter of QTH8 against seven other pathogenic fungi ranged from 21.23 mm to 31.58 mm. These results indicated that QTH8 has a broad-spectrum antimicrobial activity.

### 2.3. Effects of Bacterial Strain QTH8 on Conidial Germination

**Figure 2:**
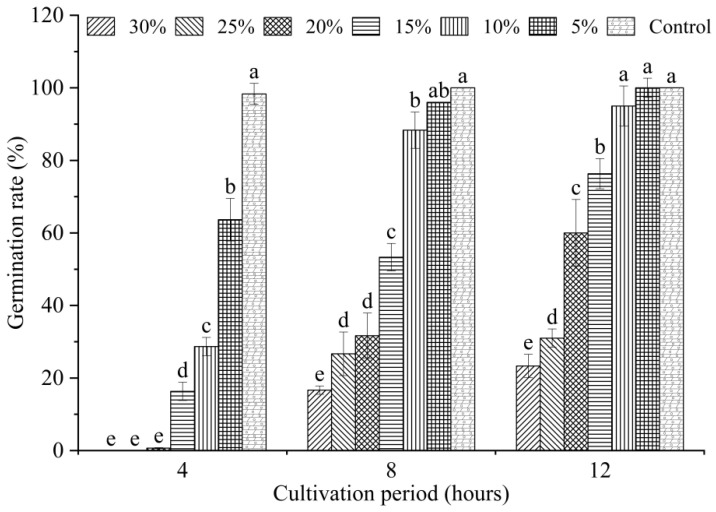
Effects of culture filtrate of bacterial strain QTH8 on conidia germination. Control: water agar medium without QTH8 supernatant. Data represent mean ± standard deviation from three repetitions. Different letters indicate a statistical difference at *p* ≤ 0.05 by a least significant difference test.

Conidial germination is a factor affecting the development and prevalence of plant pathogens. Hence, the rate of conidial germination was determined to assess the antifungal efficacy of QTH8 against *F. pseudograminearum*. We evaluated the effect of different concentrations of QTH8 culture filtrate on *F. pseudograminearum* conidial germination. Figure 2 demonstrates that six concentrations of QTH8 inhibited conidial germination, and germination rate was inclined to increase in a time-dependent manner and to decrease in a dose-dependent manner. Following 4 h of incubation, all treatments showed a significant difference in the germination rate compared with the control (*p* ≤ 0.05); however, treatment with the 25% and 30% concentration of the culture filtrates resulted in 100% inhibition of conidial germination. After 8 h exposure, treatment with the 15%, 20%, 25%, and 30% concentrations still led to a remarkable difference in the conidial germination rate in comparison with the control (*p* ≤ 0.05).

### 2.4. Active Stability of Culture Filtrates of QTH8

**Figure 3:**
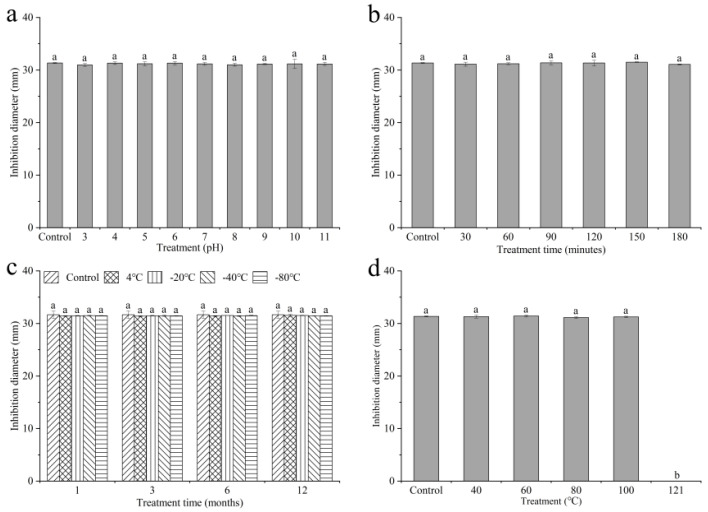
Stability of bacterial strain QTH8 culture filtrates in different conditions. (**a**) Stability of pH; (**b**) stability of UV; (**c**) stability of low temperature; (**d**) stability of high temperature. Control: original culture filtrate. Data represent mean ± standard deviation from three repetitions. Different letters indicate a statistical difference at *p* ≤ 0.05 by a least significant difference test.

To examine the stability of the antimicrobial property, culture filtrates of QTH8 were subject to pH and UV. In spite of the ‘inactivation’ treatment, the filtrate reserved similar antimicrobial efficacy against *F. pseudograminearum*, indicating that the antibacterial active substances were highly stable (Figure 3a,b). 

Culture filtrates of QTH8 exposed to various low temperatures continued to show a similar antagonistic activity to *F. pseudograminearum* (Figure 3c), indicating that the active substances are stable in low temperature conditions. 

Figure 3d shows that the antimicrobial compounds of QTH8 are stable in a certain high temperature.

### 2.5. Effect of Bacterial Strain QTH8 on Wheat Coleoptiles Infected Fusarium seudograminearum

The antagonistic activity of bacterial strain QTH8 against *F. pseudograminearum* was further tested using the coleoptiles infection assay. Two treatments significantly reduced the disease index when compared with control (Table 3), and there was a remarkable difference between Treatment 1 (wheat coleoptiles first inoculated with culture filtrates and then inoculated with *F. pseudograminearum* conidial suspension) and Treatment 2 (wheat coleoptiles first inoculated with *F. pseudograminearum* conidial suspension and then inoculated with culture filtrates). The biocontrol efficacy of Treatment 1 was 62.37 %. The two treatments showed a remarkable difference only in root length and no significant difference in plant length and fresh weight of wheat plants compared with control.

### 2.6. Effect of Bacterial Strain QTH8 on Fusarium pseudograminearum in Glasshouse

**Figure 4:**
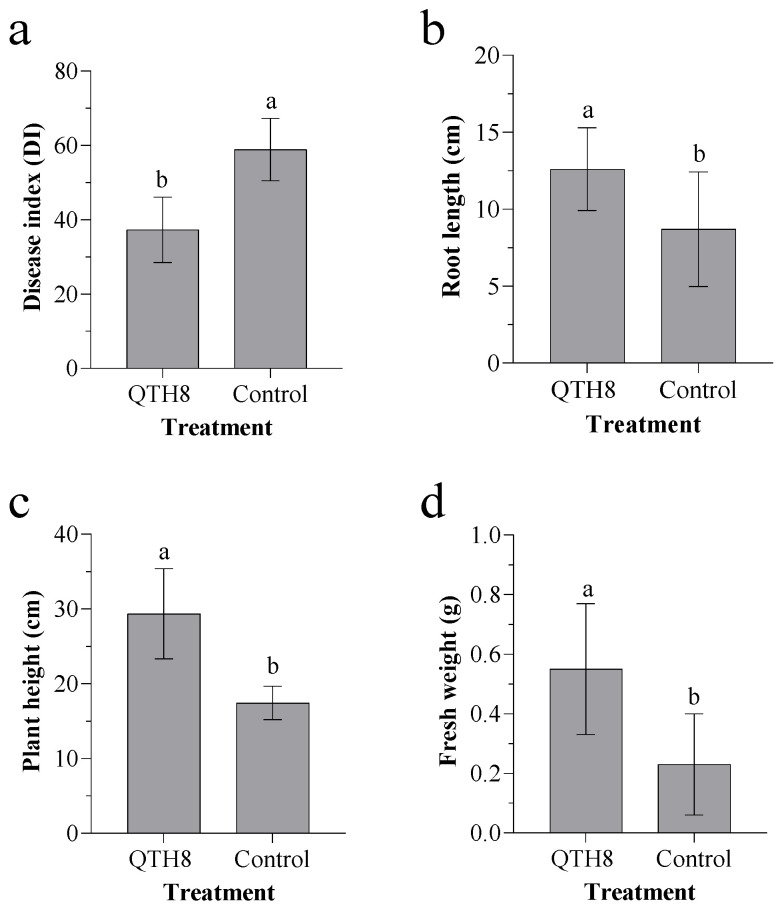
Effect of QTH8 culture filtrate treatment on: (**a**) wheat disease index, (**b**) root length, (**c**) plant height, and (**d**) fresh weight. Control: wheat plants without QTH8 culture filtrates. Data represent mean ± standard deviation from three repetitions. Different letters indicate significant difference at *p* ≤ 0.05 by a least significant difference test.

To assess the biocontrol potential of QTH8 in the glasshouse, wheat plants were bred in the presence or absence of QTH8 and *F. pseudograminearum*. The disease index was significantly lower in wheat plants treated with QTH8 than in control plants (Figure 4a). In addition, QTH8-treated wheat plants showed significantly higher root length (Figure 4b), plant height (Figure 4c), and fresh weight (Figure 4d) than control plants (*p* ≤ 0.05).

### 2.7. Identification of Bacterial Strain QTH8

**Figure 5:**
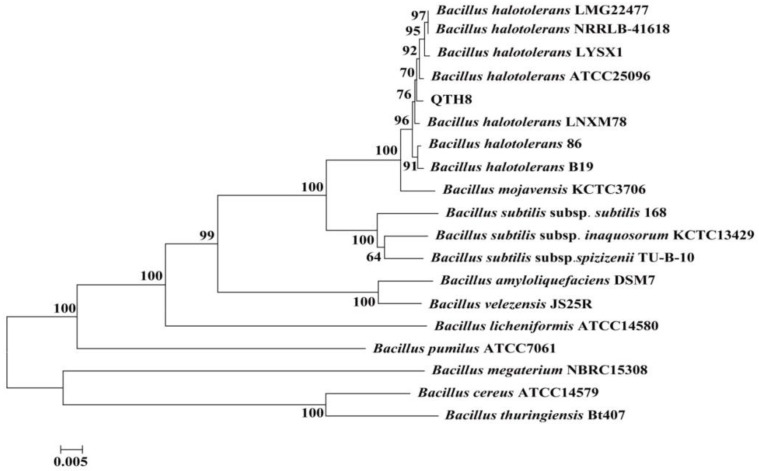
Phylogenetic tree based on concatenation of sequences of the 16S rDNA and *gyrB* genes of bacterial strain QTH8. The phylogenetic tree was constructed by the neighbor-joining (NJ) method using MEGA 5.1 software. The bootstrap values are shown at the branch points.

The phenotypic traits of QTH8 suggest that it belongs to the genus *Bacillus* (Table 4). The nucleotide sequences of the genes 16S rDNA (1439bp) and *gyrB* (1113bp) were determined and deposited in the NCBI database with accession numbers MN410608 and MN401739. The 16S rDNA and *gyrB* sequences were concatenated and compared with a connection of orthologous 16S rDNA-*gyrB* gene of *Bacillus.* By using the maximum likelihood model from MEGA 5.1 and examining the resulting trees with 1000 bootstrap replicates, bacterial strain QTH8 was determined to be associated with the cluster of *Bacillus halotolerans* (Figure 5).

### 2.8. Analysis of QTH8 Lipopeptides

**Figure 6:**
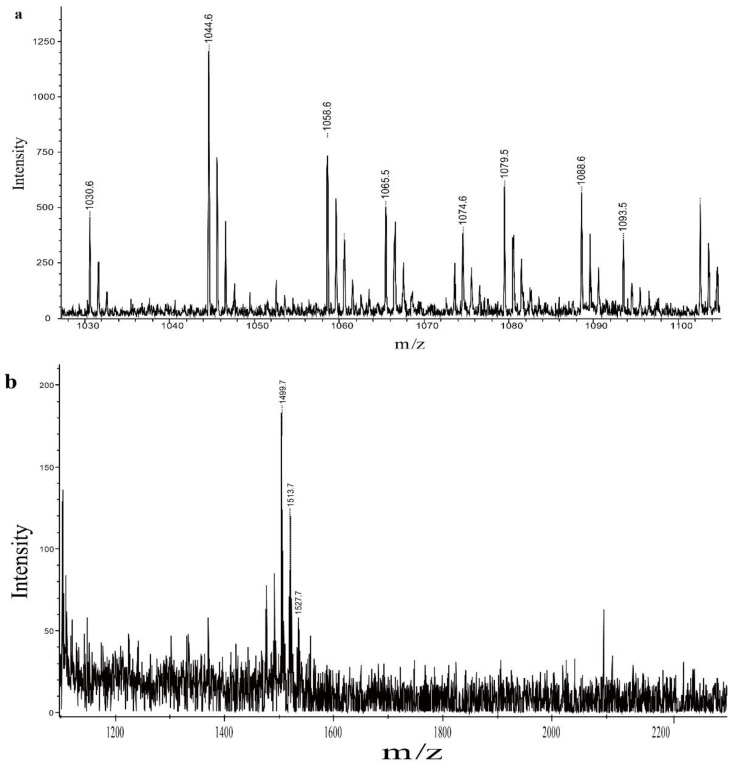
Matrix-assisted laser desorption ionization time-of-flight mass spectrometry (MALDI-TOF-MS) analysis of iturin, surfactin, and fengycin produced by bacterial strain QTH8. (**a**) iturin and surfactin; (**b**) fengycin. The MALDI-TOF-MS is recorded by Bruker Reflex MALDI-TOF.

To identify the main compounds in the crude exact, matrix-assisted laser desorption ionization time-of-flight mass spectrometry (MALDI-TOF-MS, Bruker, Saarbrucken, Germany) was used. Three lipopeptides were detected in the crude exact. There were molecular ion peaks (M + Na)^+^ for C_13_-C_15_ surfactin at *m*/*z* 1030.6, 1044.6, and 1058.6, and ion peaks (M + K)^+^ for C_15_-C_16_ surfactin at *m*/*z* 1074.6 and 1088.6 (Figure 6a). There were molecular ion peaks (M + Na)^+^ for C_14_-C_16_ iturin at *m*/*z* 1065.5, 1079.5, and 1093.5 (Figure 6a). For fengycin, there were molecular ion peaks (M + Na)^+^ for C_15_-C_17_ fengycin at *m*/*z* 1499.7, 1513.7, and1527.7(Figure 6b).

### 2.9. Functional Gene Analysis of Bacterial Strain QTH8

**Figure 7:**
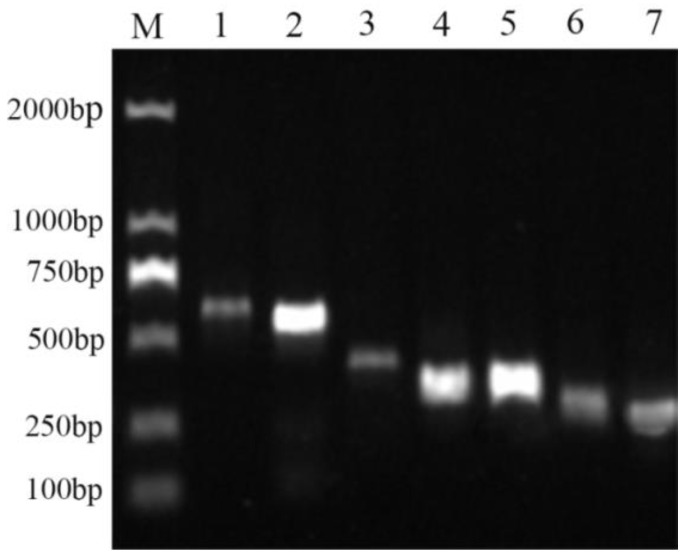
Gel electrophoresis of PCR products of lipopeptide genes of bacterial strain QTH8. Lane M, DL2000 DNA Marker; Lane 1-7, *ituC*, *bacA*, *bmyB*, *spaS*, *srfAB*, *fenD*, and *srfAA*, respectively.

To detect the lipopeptide genes of bacterial strain QTH8, PCR was performed using specific primers. The results indicated that the *srfAA*, *srfAB*, *fenD*, *spaS*, *bmyB*, *bacA*, and *ituC* genes had been amplified (Figure 7). Of these genes, the *srfAA* and *srfAB* genes encode peptide synthetases that are associated with the non-ribosomal synthesis of surfactin; *fenD* is associated with metabolites of the fengycin family; and *spaS*, *bmyB*, *bacA*, and *ituC* are associated with secondary products of the iturin family.

## 3. Discussion

Many researchers have demonstrated that culture filtrates of bacteria can control plant pathogenic fungi [27,28,29,30]. In the present study, we assessed the antagonistic efficacy bacterial strains isolated from *C. coggygria* rhizosphere soil against *F. pseudograminearum* and chosen the QTH8 strain for further analysis because of its higher antimicrobial activity. On the basis of phenotypic characteristics and phylogenetic analysis of 16S rDNA and *gyrB* genes sequence, bacterial strain QTH8 was identified as *B. halotolerans.* Previous studies have shown that *B. halotolerans* could control root rot disease of common bean and pea, verticillium wilt of cotton, grey mold disease of strawberry and tomato, plant-parasitic nematodes of tomato [14,15,16,17,18,19]. However, antimicrobial activity of *B. halotolerans* agaisnt *F. pseudograminearum* has not been reported. This study is the first, to our knowledge, to demonstrate the biocontrol efficacy of *B. halotolerans* against wheat crown rot. *B. halotolerans* QTH8 suppressed mycelial radial growth and led to mycelial malformation such as tumor formation, shortening of mycelial septum intervals, protoplast condensation, and crumpled and fractured mycelium. *B. halotolerans* QTH8 caused mycelial malformation in *H. lythri*, *Pestalotiopsis* sp., *B. cinerea*, *C. lunata*, *P. theaefolia*, *F. graminearum*, *P. nicotianae*, and *S. sclerotiorum* (data not shown). Moreover, culture filtrates of bacterial strain QTH8 demonstrated antagonistic potential against *Heterodera glycines*, *Meloidogyne javanica*, and *M. incognita* (data not shown). Similar findings have been documented by Xia et al. (2019), who found broad-spectrum activity of *B. halotolerans* LYSX1 against diverse plant pathogens [31].

Conidia play an important part in the initial infection stage of crown rot diseases. Many studies have documented that *Bacillus* spp. reduces sporangium germination and affects mycelial growth [32,33,34]. The present study showed similar results. The coleoptile infection assay used in this study also suggested that *B. halotolerans* QTH8 is capable of preventive control against wheat crown rot.

Owing to their wide-spectrum antagonistic activity, various bacterial strains have been used to manage crop diseases [9,35,36]. Nevertheless, to our knowledge, the control of wheat crown rot using *B. halotolerans* has not been documented. The outcomes from the glasshouse environment indicated that the application of QTH8 improved the growth parameters of wheat seedlings (root length, plant height, and fresh plant weight) and reduced the severity of the disease caused by *F. pseudograminearum*. Several research groups have reported similar findings [17,18,19,31]. However, it is essential to verify the potential of QTH8 against *F. pseudograminearum* under field tests in future research.

*Bacillus* species produce various secondary metabolites including bacteriocins, fengycin, surfactin, iturin, bacillibactin, acetoin, and so on [37,38,39]. These biological active substances have huge potential for applications to control plant diseases in sustainable agricultural ecosystems. Analysis of the QTH8 culture filtrates demonstrated that the antimicrobial substances were pH and UV stable, cold-resistant, and stable up to a certain high temperature. Unpublished data indicate that lipopeptides obtained from QTH8 culture filtrates controlled *F. pseudograminearum* in in vivo and in vitro trials, but the effect was lower when compared with that of QTH8 culture filtrates. These findings indicate that culture filtrates of QTH8 contained antimicrobial active compounds other than lipopeptides.

MALDI-TOF-MS is a rapid, accurate, and low-cost method, extensively used to examine bioactive secondary compounds [40,41]. Many researchers have used MALDI-TOF-MS to detect antimicrobial active compounds produced by bacteria [41,42]. In the present study, secondary metabolites of QTH8 were classified into three families—surfactin, iturin, and fengycin—according to the MALDI-TOF-MS analysis. Different hosts, locations, and biotopes may lead to the production of different lipopeptide antibiotics by *B. halotolerans*. For example, *B. halotolerans* BT5 has been reported to produce surfactin and fengycin, whereas *B. halotolerans* PVB16 has been described to produce iturin and fengycin [15,17]. Manifold antibiotic substances—including fengycins, surfactins, iturins, bacilysins, rhizocticins, and amicoumacins—have been detected in *B. subtilis* culture filtrates [40]. Koumoutsi et al. (2004) have reported that *B. amyloliquefaciens* FZB 42 could produce three antifungal active compounds including surfactin, bacillomycin D, and fengycin [41]. Genes—such as *bmyB*, *fenD*, *ituC*, *srfAA*, and *srfAB*—encode and regulate these active substances in the antimicrobial peptide biosynthetic pathways. These marker genes have previously been used to test the presence of lipopeptide compounds of many *Bacillus* species [43,44]. Furthermore, *Bacillus* strains possessing more marker genes were shown to be more effective at controlling plant diseases than *Bacillus* strains lacking one or more of these marker genes [45]. In the present study, results indicated the presence of *srfAA*, *srfAB*, *fenD*, *spaS*, *bmyB*, *bacA*, and *ituC* in *B. halotolerans* QTH8. These genes encode surfactin (*srfAA* and *srfAB*), fengycin (*fenD*), and iturins (*spaS*, *bmyB*, *bacA*, and *ituC*), respectively. These findings are consistent with the findings of the MALDI-TOF-MS analysis. The results demonstrated that *B. halotolerans* QTH8 produced various biologically active substances that can control various plant diseases. Further research is necessary to assess the antimicrobial compounds and elucidate the antagonistic mechanism of QTH8.

In conclusion, *B. halotolerans* QTH8 is a vital candidate in managing wheat crown rot. We also showed that bacterial strain QTH8, which harbors multiple antimicrobial peptide marker genes, has the potential to control other crop diseases.

## 4. Materials and Methods

### 4.1. Fungal Isolates, Bacterial Strains, and Growth Conditions

The fungal isolates used in this study (Table 5) were cultured at 25 °C. The bacterial strains were isolated from *C. coggygria* rhizosphere soil in Jiaozuo, Henan Province, China according to the procedure described by Yang et al. (2014) [46], and stored in this lab. Luria broth (LB) was used for culturing *Bacillus* spp. at 37 °C. Landy culture medium was used for fermenting the bacterial strains [47]. Potato saccharose agar (PSA) medium was used for culturing the fungal isolates.

### 4.2. Anagonism of Bacterial Strains against Fusarium pseudograminearum In Vitro

A dual culture method was used to test the antagonism of bacteria against *F. pseudograminearum* [48,49]. Fermented cultures of obtained bacterial strains were collected and sterilized through a 0.22 μm filter. A freshly fungal mycelial block (5 mm) was placed at the center of a PSA plate, and 5 μL culture filtrates of isolates were placed at 25 mm away from the mycelial block. A PSA plate with fungal mycelial blocks was used as the control. Plates in all treatments were incubated at 26 °C for 5 days, and the inhibition diameter was measured. Tests were performed three times with three replicates per treatment.

### 4.3. Determination of Antimicrobial Spectrum of Bacterial Strain QTH8 Culture Filtrates

The dual culture method was also used to detect the antimicrobial activity of bacterial strain QTH8 against *S. sclerotiorum*, *F. graminearum*, *C. lunata*, *B. cinerea*, *P. nicotianae*, *H. lythri*, *Pestalotiopsis* sp., and *P. theaefolia* as the previous description. A PSA plate with fungal mycelial blocks was used as a control. Tests were performed three times with three replicates per treatment.

### 4.4. Effects of Bacterial Strain QTH8 Culture Filtrate on Conidia Germination of Fusarium pseudograminearum

A fresh block of *F. pseudograminearum* was cultured in mung bean soup medium at 25 °C and incubated in a shake at 1.6× *g* for 5 days. Conidia were collected at 17, 896× *g* for 5 min, and the suspension concentration was adjusted to 1 × 10^6^ conidia/mL using a hemocytometer. Then, water agar medium (WA) plates treated with 5%, 10%, 15%, 20%, 25%, and 30% QTH8 culture filtrates was inoculated with 100 µL *F. pseudograminearum* conidial suspensions. An untreated WA plate was used as a control. Conidial germination was counted as described previously [50]. Tests were performed three times with three replicates per treatment.

### 4.5. Treatment of QTH8 Culture Filtrates

The pH sensitivity of QTH8 was detected by separately adjusting the pH to 3.0, 4.0, 5.0, 6.0, 7.0, 8.0, 9.0, 10.0, and 11.0, and testing the antimicrobial activity using the previous procedure.

The UV resistance of the metabolites of QTH8 was separately tested by exposing the culture filtrates to a 35 cm UV light source for 30, 60, 90, 120, 150, and 180 min and subsequently examining the antagonistic activity of treated culture filtrates according to the method described above.

To test the heat resistance, QTH8 culture filtrates were incubated at 40, 60, 80, and 100 °C in a water bath for 10 min or subjected to hot pressing sterilization at 121 °C for 10 min; then the antimicrobial activity was tested as described above.

The cold stability of culture filtrates was assessed by storing them at 4, −20, −40, and −80 °C for 1, 3, 6, and 12 months, and their antimicrobial activity was tested.

All the above experiments were repeated three times with three replicates per treatment.

### 4.6. Assessment of the Effect of QTH8 Culture Filtrates on Fusarium pseudograminearum-Infected Seedlings by Coleoptile Inoculation

Wheat seedlings (zhengmai 366) cultured for 3 days were selected for seedling trials based on previously described procedures [51]. In brief, the apical portion of wheat coleoptiles was excised. Two treatments were used to assess the effect of QTH8: (1) coleoptiles were inoculated at the wound site first with culture filtrates (500 μL) and after 12 h exposure inoculated with *F. pseudograminearum* conidial suspension (3 μL); (2) coleoptiles were inoculated at the wound site first with *F. pseudograminearum* conidial suspension and after 12 h exposure inoculated with culture filtrates. All treated seedlings were incubated at 26 °C in a glasshouse for 7 days. Disease index and plant parameters were analyzed as described by Bovill et al. (2010) [52]. Tests were performed three times with three replicates per treatment.

### 4.7. Antagonism of Bacterial Strain QTH8 against Fusarium pseudograminearum In Vivo

Fresh mycelium was inoculated on sterilized millet medium and incubated at 25 °C until the mycelium completely covered the medium. The millet culture was mixed with autoclaved sandy loamy soil (1:1 *v*/*v*) to obtain the diseased substrate, and 200 g of the diseased substrate was placed into a plastic pot with a 9.5 cm diameter and 12 cm height. Wheat seeds (2 g) were sterilized with 75% ethanol for 1 min, and rinsed three times with sterile water, and then mixed with 1 mL of QTH8 culture filtrates. Seven wheat seeds were cultivated in the plastic pot with 30 g diseased substrate. Untreated wheat seeds without the addition of culture filtrates were used as a control. The experiment was performed thrice, and each treatment consisted of three replicates, which were randomly cultured in the glasshouse. At 21 days post-inoculation, disease index and plant parameters of wheat seedlings were analyzed as described previously [2,53].

### 4.8. Identification of QTH8

The phenotypic characteristic of bacterial strain QTH8 was tested according to the described method [54].

Genomic DNA was extracted using the Bacterial Genomic DNA Extraction Kit (OMEGA Bio-Tek, China) according to the manufacturer’s protocol. 16S rDNA and *gyrB* genes were amplified using specific primer pairs, respectively (Table 6). PCR products were sequenced, and the nucleotide sequences were submitted to the NCBI nucleotide sequence database. According to the sequencing results, the gene sequences of related strains were downloaded from GenBank, the 16S rDNA and *gyrB* gene sequences were concatenated, multiple sequence alignment was performed by ClustalX, and a phylogenetic tree was constructed using MEGA5.1 software using neighbor-joining (NJ) method [55]. The stability of the phylogenetic tree was analyzed by bootstrapping with 1000 replicates.

### 4.9. Identification of Lipopeptides

Culture filtrates were calibrated at pH 2.0 with 6 M hydrochloric acid and were placed in a refrigerator at 4 °C for 24 h. The precipitates were collected by centrifugation at 7155× *g* for 10 min at 4 °C, and dissolved in methanol. The pH of the solution was adjusted to 7.0 with 2.0 M sodium hydroxide, and then the solution was evaporated to dryness in a rotary evaporator to obtain a powder of lipopeptide extract. The crude extract of lipopeptides was dissolved in methanol (10 mg/mL) and stored at 4 °C for subsequent use [56].

The compositional analysis of the lipopeptides of bacterial strain QTH8 was performed as described in previous studies [41,45]. The crude extracts of lipopeptides collected in the above method were filtered using a 0.22-μm bacterial filter. The active components were analyzed by MALDI-TOF-MS, recorded on a Bruker Reflex MALDI-TOF instrument, using a 337 nm nitrogen laser for desorption and ionization with a matrix of α-cyano-4-hydroxycinnamic acid.

### 4.10. Detection of Antimicrobial Peptide Biosynthetic Genes

The primer pairs for the amplification of the antimicrobial peptide biosynthetic genes are listed in Table 6. The primer sequences were obtained from previous studies [33,54]. The PCR outcomes were tested by gel electrophoresis.

### 4.11. Statistical Analysis

Data were recorded and analyzed in DPS v9.01 software (Zhejiang University, Hangzhou, China). The one-way analysis of variance (ANOVA) was used to analyze the experimental data, and the least-significant difference (LSD) was used to test the different significance in the level of *p* ≤ 0.05.

## Figures and Tables

**Table 1:** Effect of five bacterial strains against *Fusarium pseudograminearum*.

Strain	Inhibition Diameter (mm)
QTH8	31.67 ± 0.68 a
QTH2	28.61 ± 0.49 b
QTH5	25.23 ± 0.25 d
QTH1	22.37 ± 0.26 e
QTH7	19.53 ± 0.31 f

Data represent mean ± standard deviation from three repetitions. Different letters within a column indicate a statistical difference at *p* ≤ 0.05 by a least significant difference test.

**Table 2:** Effect of bacterial strain QTH8 on eight phytopathogenic fungi.

Phytopathogenic Fungi	Inhibition Diameter (mm)
*Hainesia lythri*	34.09 ± 0.14 a
*Pestalotiopsis* sp.	31.58 ± 0.95 b
*Botrytis cinerea*	31.25 ± 0.64 b
*Curvularia lunata*	31.03 ± 1.03 b
*Phyllosticta theaefolia*	30.98 ± 1.45 b
*Fusarium graminearum*	30.97 ± 0.68 b
*Phytophthora nicotianae*	21.27 ± 0.05 c
*Sclerotinia sclerotiorum*	21.23 ± 1.45 c

Data represent mean ± standard deviation from three repetitions. Different letters within a column indicate a statistical difference at *p* ≤ 0.05 by a least significant difference test.

**Table 3:** Effect of bacterial strain QTH8 on coleoptiles of wheat seedlings infected with *Fusarium pseudograminearum*.

Treatment	Root Length (cm)	Plant Height (cm)	Fresh Weight (g)	Disease Index	Efficacy (%)
Control	15.49 ± 1.12 c	12.87 ± 1.50 a	0.18 ± 0.01 a	70.24 ± 3.76 a	
Treatment 2	18.57 ± 1.96 b	13.45 ± 1.87 a	0.17 ± 0.01 a	58.82 ± 6.32 b	16.26
Treatment 1	21.73 ± 0.38 a	12.20 ± 0.41 a	0.18 ± 0.01 a	26.43 ± 5.18 c	62.37

Control: coleoptiles inoculated with *F. pseudograminearum* conidial suspension; Treatment 1: coleoptiles first inoculated with culture filtrates and then with conidia suspension; Treatment 2: coleoptiles first inoculated with *F. pseudograminearum* conidial suspension and then with culture filtrates. Data represent mean ± standard deviation from three repetitions. Different letters within a column indicate a statistical difference at *p* ≤ 0.05 by a least significant difference test.

**Table 4:** Physiological and biochemical properties of bacterial strain QTH8.

	Result	Characteristic	Result
Shape	rod	Acid from:	
Endospore	+	D-Glucose	+
Gram stain	+	D-Mannitol	+
Citrate utilization	+	Glycerine	+
Nitrate reduction	+	Sucrose	+
MR reaction	−	Lactose	−
Indole production	−	Gas from:	
Starch hydrolysis	+	D-Glucose	−
Casein hydrolysis	+	D-Mannitol	−
Anaerobic growth	−	Glycerine	−
V-P test	+	Sucrose	+
Catalase test	+	Lactose	−
Litmus milk test:		Growth at:	
Acid reaction	−	5 °C	+
Alkaline reaction	−	10 °C	+
Curd formation	−	20 °C	+
Peptonization	+	45 °C	+
Growth in 10% NaCl	+	50 °C	−

“+”and “−” indicate positive and negative reactions, respectively.

**Table 5:** Pathogenic fungal strains used in this study.

Isolates	Plant Disease	Sources
*Fusarium pseudograminearum*	Wheat crown rot	This lab
*Hainesia lythri*	Peony coelomycete leaf spot	This lab
*Pestalotiopsis* sp.	Moonflower leaf spot	This lab
*Botrytis cinerea*	Tomato gray mold	This lab
*Curvularia lunata*	Maize leaf spot	This lab
*Phyllosticta theaefolia*	Tea white scab	This lab
*Fusarium graminearum*	Fusarium head blight	This lab
*Phytophthora nicotianae*	Tobacco black shank	This lab
*Sclerotinia sclerotiorum*	Rape sclerotinia stem rot	This lab

**Table 6:** Primers used in this study.

Primer	Sequence (5′-3′)	Use of Primers
*bmyB*-F	TGAAACAAAGGCATATGCTC	*bmyB* gene amplification
*bmyB*-R	AAAAATGCATCTGCCGTTCC	
*ituC*-F	TTCACTTTTGATCTGGCGAT	*ituC* gene amplification
*ituC*-R	CGTCCGGTACATTTTCAC	
*srfAB*-F	GTTCTCGCAGTCCAGCAGAAG	*srfAB* gene amplification
*srfAB*-R	GCCGAGCGTATCCGTACCGAG	
*srfAA*-F	GAAAGAGCGGCTGCTGAAAC	*srfAA* gene amplification
*srfAA*-R	CCCAATATTGCCGCAATGAC	
*spaS*-F	GGTTTGTTGGATGGAGCTGT	*spaS* gene amplification
*spaS*-R	GCAAGGAGTCAGAGCAAGGT	
*bacA*-F	CAGCTCATGGGAATGCTTTT	*bacA* gene amplification
*bacA*-R	CTCGGTCCTGAAGGGACAAG	
*fenD*-F	CCTGCAGAAGGAGAAGTGAAG	*fenD* gene amplification
*fenD*-R	TGCTCATCGTCTTCCGTTTC	
27F	AGAGTTTGATCMTGGCTCAG	16S rDNA gene amplification
1492R	GGYTACCTTGTTACGCACTT	
*gyrB* 1	GAAGTCATCATGACCGTTCTGCAYGCNGGNGGNAARTTYGA	*gyrB* gene amplification
*gyrB* 2	AGCAGGGTACGGATGTGCGAGCCRTCNACRTCNGCRTCNGTCAT	

## Data Availability

Not applicable.
